# Construction of a fusion plasmid containing the PSCA gene and cytotoxic T-lymphocyte associated antigen-4 (CTLA-4) and its anti-tumor effect in an animal model of prostate cancer

**DOI:** 10.1590/1414-431X20165620

**Published:** 2016-10-24

**Authors:** T.J. Mai, R. Ma, Z. Li, S.C. Bi

**Affiliations:** Department of Urology, China Meitan General Hospital, Beijing, China

**Keywords:** Prostate cancer, DNA vaccine, PSCA, CTLA-4

## Abstract

Cytotoxic T lymphocyte-associated antigen-4 (CTLA-4) is a negative regulator of T cell activation, which competes with CD28 for B7.1/B7.2 binding, and which has a greater affinity. Fusion of specific antigens to extracellular domain of CTLA4 represents a promising approach to increase the immunogenicity of DNA vaccines. In this study, we evaluated this interesting approach for CTLA4 enhancement on prostate stem cell antigen (PSCA)-specific immune responses and its anti-tumor effects in a prostate cancer mouse model. Consequently, we constructed a DNA vaccine containing the PSCA and the CTLA-4 gene. Vaccination with the CTLA4-fused DNA not only induced a much higher level of anti-PSCA antibody, but also increased PSCA-specific T cell response in mice. To evaluate the anti-tumor efficacy of the plasmids, murine models with PSCA-expressing tumors were generated. After injection of the tumor-bearing mouse model, the plasmid carrying the CTLA4 and PSCA fusion gene showed stronger inhibition of tumor growth than the plasmid expressing PSCA alone. These observations emphasize the potential of the CTLA4-fused DNA vaccine, which could represent a promising approach for tumor immunotherapy.

## Introduction

Prostate cancer is one of the most frequently diagnosed cancers, and is the second leading cause of cancer mortality among males in the United States of America (1). It has also become an increasingly important health problem in China. Surgery and radiation are the treatments of choice only for early stage (localized) prostate cancer. There is yet no effective treatment for patients who develop recurrences or for those who have metastatic disease at the time of diagnosis (2). Advances in molecular and immune biology allow for the potential use of tumor vaccines as immune therapy. In the last decade, a variety of vaccines against prostate cancer have been developed and tested in clinical trials for safety and therapeutic profile (3).

DNA vaccines are made up of eukaryotic expression plasmids that carry an antigen of interest. When the DNA vaccine is injected into the body of an organism, the foreign target antigen is expressed *in vivo*, which activates the body's immune system, inducing specific humoral and cellular immune responses (4). In recent years, rapid progress has been made in the development of DNA vaccines, particularly in those that are used for the treatment of cancer and chronic infectious diseases (5). Unfortunately, the immunogenicity of plasmid DNA in humans has proven to be modest compared with the immunogenicity observed in other species treated with microbial expression vectors. Fusion of antigens to extracellular domain of cytotoxic T lymphocyte-associated antigen-4 (CTLA-4) has proven to be a potential approach to enhance the immunogenicity of DNA vaccine, especially in large animals (6). Many scholars demonstrated that a fusion DNA vaccine containing the extracellular domain of CTLA-4 was able to induce both robust antibody response and T cell response (7–9).

Recent studies have found that levels of prostate stem cell antigen (PSCA) have higher diagnostic and prognostic value in prostate cancer compared with prostate-specific antigen (10). PSCA is a newly discovered tumor-associated antigen with high specific expression in various types of prostate cancer and metastatic cancer, and its expression is not reduced with tumor progression. Additionally, PSCA protein anchors to the surface of cancer cells by a GPI without exocytosis, so it is considered an ideal target antigen for prostate cancer immunotherapy (11). In this study, we evaluated this interesting approach for the enhancement on PSCA-specific immune responses and its anti-tumor effects in a prostate cancer mouse model.

## Material and Methods

### Cell lines and mice

The mouse prostate tumor cell line RM-1 was purchased from Shanghai Cell Institute (Shanghai, China). To generate a cell line that stably expressed human PSCA (GenBank: AJ297436.1), RM-1 were transfected with a plasmid carrying PSCA (pcDNA3.1-PSCA), and then the cells were subjected to selection by treatment with 800 μg/mL G418. This cell line is hereafter referred to as RM-1-PSCA. The expressing of PSCA in these cells was demonstrated by western blot. Briefly, RM-1-PSCA cells were treated with RIPA lysis buffer before electrophoresis, and processed in 12% SDS-PAGE (Bio-Rad, USA) under reducing conditions for western blotting. Immunoblot analysis was carried out with the mouse mAb to human PSCA (Sigma, USA) as the primary antibody; HRP-labeled goat anti-mouse IgG (Sigma) was used as the secondary antibody for human PSCA and results were visualized with enhanced chemiluminescence.

Female C57BL/6 mice, aged 4-6 weeks, were purchased from Beijing Weitong Lihua Experimental Animal Technology Co. Ltd. (China). All animal care and experimental procedures were approved by the Institutional Animal Care and Use Committee of the China Meitan General Hospital.

### Construction and expression of CTLA-4 fusion DNA vaccines

Based on the coding regions of PSCA (GenBank: AJ297436.1) and CTLA-4 (GenBank: BC074893.2), we designed a PSCA (coding gene 20-391) and CTLA-4 (coding gene 32-703) fusion gene. The PSCA gene was connected to the CTLA-4 gene by Furin-2A (F2A). Synthesis of the fusion gene was carried out by Taihe Gene, China. The fusion gene was cloned into the expression vector pVAX1.

The resulted plasmid pVAX1-PSCA-F2A-CTLA-4 was transiently transfected into 293T cells (Shanghai cell bank of Chinese Academy of Sciences) by Lipofectamine 2000™ (Invitrogen, USA, 11668019), and flow cytometry was performed to confirm the validity of this construct. Briefly, 1×10^6^ of 293T cells were collected 48 h after transfection. After washing with buffer (cold PBS, 2% BSA), the cells were treated with a FITC-labeled mouse anti-PSCA antibody (Santa Cruz Biotechnology, USA, sc-80654 FITC), as well as with a Phycoerythrin (PE)-labeled mouse anti-CTLA-4 (Santa Cruz Biotechnology, sc-376016 PE). The cells were next analyzed by flow cytometry using the Cell Quest software package (Becton-Dickinson FACScalibur, USA).

### DNA vaccination and *in vivo* tumor treatment experiments

The experimental mice were randomly divided into 4 groups (6 mice per group): the untreated group, the pVAX1 group, the pVAX1-PSCA group and the pVAX1-PSCA-F2A-CTLA-4 group. At the same time of the first vaccination, the mice were subcutaneously inoculated with RM-1-PSCA cells (1.0×10^5^) in the right flank back. Each mouse was injected with 100µg plasmid and subjected to electroporation. On the 10th and 20th day after the first immunization, the mice were given a boost dose. Tumor measurement was performed using calipers, and tumor volume was calculated according to the following formula: V (mm^3^) = 0.5 × long diameter × (short diameter)^2^. Based upon tumor volume calculations, growth curves of the tumors were plotted. The experiments were independently repeated three times.

### Detection of serum antibodies

ELISA plates (96-well) were prepared by coating with PSCA at 1 μg/mL in PBS buffer, 4°C overnight, and then blocked with 5% powdered milk in PBS containing Tween-20. Mouse sera taken 2 weeks after the final immunization were serially diluted in PBS and incubated in the plates for 2 h at 37°C. Then, the plates were incubated with HRP-conjugated goat anti-mouse IgG diluted 1:2500 for 2 h at room temperature. The absorbance was measured at 405 nm on an ELISA reader.

IgG subclasses were also evaluated by ELISA. Goat anti-mouse IgG1 or goat anti-mouse IgG2a coupled to HRP was used for detecting the IgG subclasses. The cutoff value was set as two times greater than that of the negative controls.

### ELISPOT assay and cytokine secretion assay

Two weeks after the final vaccination, splenocytes from the control and vaccinated groups of mice were isolated. An IFN-γ enzyme-linked immunospot (ELISPOT) assay was performed according to the manufacturer’s protocol (Dakewe Biotech Ltd., China). In brief, 96-well plates were coated with an anti-mouse interferon (IFN)-γ monoclonal antibody (mAb) at 4°C overnight and then blocked for 1 h at 37°C. Freshly isolated splenocytes (4×10^5^ cells/well) from each vaccinated mouse group were added to the wells and incubated with 10 μg/mL of a recombinant PSCA for 36–48h. Each test condition was performed in triplicate. The spots were counted and analyzed with the ELISPOT Reader.

The amounts of IFN-γ and IL-4 produced by splenocytes in the ELISPOT assay were quantified using an ELISA kit (Sigma-Aldrich Corporation, China), and carried out according to the manufacturer’s instructions.

### Statistical analysis

Differences between groups were analyzed by one-way analysis of variance using the SPSS 17.0 software (USA). A P value of less than 0.05 was considered to be statistically significant.

## Results

### Expression of PSCA in RM-1 cells and of fusion construct in 293T cells

**Figure 1:**
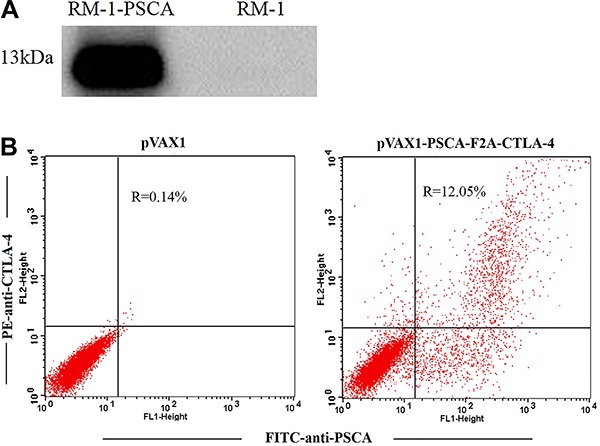
Expression of prostate stem cell antigen (PSCA) in RM-1-PSCA cells and fusion DNA vaccine in 293T cells by western blot (*A*). *Left*, lysates of RM-1 cells stably transfected with pcDNA3.1-PSCA; *Right*, lysates of non-transfected RM-1 cells. *B*, expression of plasmid pVAX1-PSCA-F2A-CTLA-4 was studied by flow cytometry. *Left*, 293T cells transfected with pVAX1; *Right*, 293T cells transfected with pVAX1-PSCA-F2A-CTLA-4.

Western blot analysis, using a primary Ab against human PSCA, confirmed the expression of PSCA in RM-1-PSCA cells (Figure 1A). A single band of the PSCA protein with an approximate molecular weight of 13 kDa was observed, in agreement with the expected molecular weight.

The expression of plasmid pVAX1-PSCA-F2A-CTLA-4 was studied by flow cytometry. The pVAX1-PSCA-F2A-CTLA-4 group showed positive results, while in the negative controls no positive cells were detected. The coexpression rate of PSCA and CTLA-4 in the transfected 293T cells was 12.05% (Figure 1B).

### Mice immunized with fusion DNA vaccine generated both enhanced humoral and cellular specific immune responses

**Figure 2:**
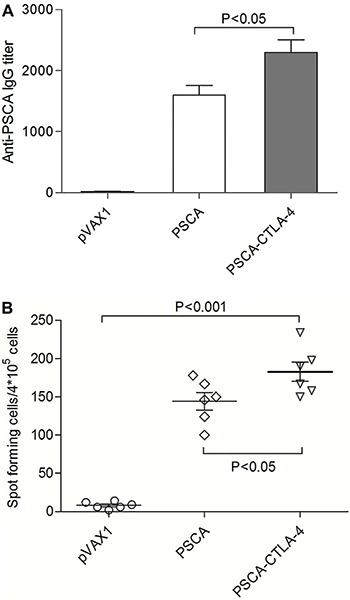
*A*, specific antibody response induced by DNA vaccines. Groups of experimental mice were immunized with the pVAX1, pVAX1-PSCA, or pVAX1-PSCA-CTLA-4 plasmids. Anti-prostate stem cell antigen (PSCA) antibodies were determined by ELISA after immunization. *B*, Analysis of the spot frequencies of antigen-specific IFN-γ T cells. Splenocytes from vaccinated mice were harvested 2 weeks after final immunization. Data are reported as means±SD (ANOVA).

In order to develop novel therapeutics specifically targeting CTLA-4, we constructed a DNA vaccine by cloning the sequence of human CTLA-4 fused with PSCA gene into pVAX1. After immunization of mice by intramuscular injection once every 10 days for three times, the mice serum was collected and examined for specificity to PSCA. Our data indicates that this DNA vaccine can induce antibody specific to PSCA (Figure 2A). The recombinant plasmid pVAX1-PSCA-F2A-CTLA-4 induced antibody levels that were approximately 1.5 times that of the pVAX1-PSCA group. The antibody titers of mice vaccinated with pVAX1-PSCA-F2A-CTLA-4 were significantly higher than the other groups (P<0.05). This experiment showed that the fusion of CTLA-4 and PSCA could significantly improve the antibody response induced by a DNA vaccine.

As shown in Figure 2B, the number of spots detected in the ELISPOT assay, using cells from the pVAX1-PSCA-F2A-CTLA-4 vaccinated groups, was significantly higher than in cells from the pVAX1 controls (P<0.001) and than in cells from mice immunized with pVAX1-PSCA (P<0.05).

### Augmentation of both Th1 and Th2 responses

**Figure 3:**
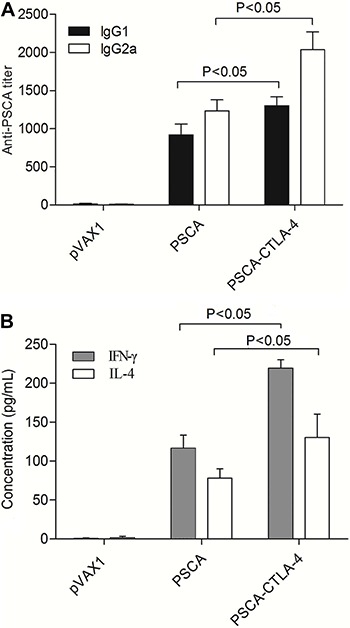
DNA vaccine induced antigen-specific Th1 and Th2 immune responses analyzed in sera from mice at 2 weeks after the final immunization. *A*, antibody subtype (IgG1 and IgG2a) responses. *B*, cytokine profile of proliferating T cells. Data are reported as means±SD (ANOVA).

Since the quality of the immune response is crucial for the efficacy of any vaccine, we evaluated the immune response polarization by analyzing the antibody subclasses generated. PSCA-specific IgG was subtyped by ELISA using IgG1 and IgG2a antibodies in mice vaccinated with groups of plasmids. Figure 3A shows that the series of constructs could mediate both PSCA-specific IgG2a (Th1) and IgG1 (Th2) antibody responses. The level of IgG2a and IgG1 in the pVAX1-PSCA-F2A-CTLA-4 group was significantly higher than in the group of pVAX1-PSCA (P<0.05).

To further characterize the polarization of the immune response, IFN-γ and IL-4 levels were also measured as an indication of the Th1 and Th2 cell response, respectively. As shown in Figure 3B, splenocytes of mice immunized with pVAX1-PSCA-F2A-CTLA-4 exhibited greatly increased secretion of the Th1 (IFN-γ) and Th2 cytokines (IL-4) compared with the pVAX1-PSCA group (P<0.05).

### Enhancement of suppressed growth of PSCA-expressing RM-1 cell in CTLA-4 fusion DNA immunized mice

**Figure 4:**
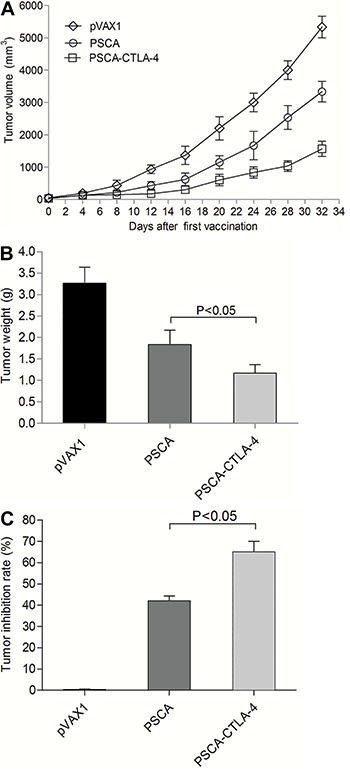
Anti-tumor efficacy of DNA vaccines in a tumor bearing mouse model after immunization. The mice were subcutaneously inoculated with RM-1-PSCA cells (1.0×10^5^) in the right flank back. *A*, tumor growth curve; *B*, mean tumor weight; *C,* tumor growth inhibition rate. Data are reported as means±SD (ANOVA).

To examine the protective effect of immunity against PSCA and CTLA-4, we subcutaneously inoculated the C57BL/6 mice with RM-1-PSCA cells. The RM-1-PSCA tumor growth rate in the pVAX1-PSCA-F2A-CTLA-4-vaccinated mice was lower than that in the mice treated with the control plasmid pVAX1 (Figure 4A). Moreover, the pVAX1-PSCA-F2A-CTLA-4 vaccine inhibited tumor growth more effectively than pVAX1-PSCA vaccine (P<0.05).

We also measured tumor weight, and calculated tumor growth inhibition rates in mice after treatment with different plasmid vaccines. Tumor weight in the mice immunized with pVAX1-PSCA-F2A-CTLA-4 was significantly lower compared to that in mice immunized with pVAX1-PSCA (P<0.05; Figure 4B). Similarly, tumor growth inhibition rate in mice immunized with pVAX1-PSCA-F2A-CTLA-4 was significantly higher than that in mice immunized with pVAX1-PSCA (P<0.05; Figure 4C).

## Discussion

Prostate cancer is the most commonly diagnosed malignancy in men in the United States, and is second in cancer-related deaths only surpassed by lung cancer, with 29,480 projected deaths in 2014 (12). Although recent years have seen great advances in treatments for prostate cancer, including second-line chemotherapy, anti-androgen therapies, and radiopharmaceuticals, none of these therapies are curative (13). Nonetheless, there is great potential for these and other existing therapies to be used synergistically with immunotherapies already present in clinical practice or in late stages of clinical trials. Furthermore, given the lack of significant toxicity seen with therapeutic cancer vaccines and the lack of over-lapping toxicity seen with immune checkpoint inhibitors, it appears possible that immune-based combinations have the potential for improving clinical outcomes without causing patients significant additional side effects. This is very important in a disease such as prostate cancer where symptoms from the disease are generally not present until the late stages (14). However, many studies have indicated that a vaccine containing only an antigen is not effective enough to induce an anti-tumor immune response (4,15).

An important area of research is aimed at improving the immunogenicity of DNA vaccines by way of molecular adjuvants. Fusion of antigens to extracellular domain of CTLA-4 has been proven to be a potential approach to enhance the immunogenicity of DNA vaccine, especially in large animals (16). Meanwhile, the selection of the target antigen has a vital role in the specificity and effectiveness of treatment. PSCA is a recently identified target antigen that is expressed in the cell membrane rather than secreted (17). Because of its high level of expression in all stages of prostate cancers and metastases, PSCA is a target antigen in prostate cancer treatment (18). Immune therapy using PSCA as the target antigen has been included in passive immunotherapy, such as in the use of monoclonal antibodies, and in active immunotherapy, such as in vaccines, which include protein vaccines, DC cell vaccines and DNA vaccines (19).

In the present study, we evaluated the interesting approach for CTLA-4 enhancement on PSCA-specific immune responses and its anti-tumor effects in a mouse model. Compared with the antigen only control group, vaccination with the CTLA4-fused DNA not only induced a much higher level of anti-PSCA antibody, but also it increased PSCA-specific T cell response in mice, which was in line with our expectations. This is mainly because CTLA4-fused DNA vaccine can induce anti-CTLA-4 antibodies, thus avoiding competitive binding of B7 with CD28. So, the inhibitory effect of CTLA-4 on humoral immune response and cellular immune response was eliminated.

To evaluate the anti-tumor efficacy of the plasmids, murine models with PSCA-expressing tumors were generated. After injection of the tumor-bearing mouse model with DNA vaccine, the plasmid carrying the fusion gene of CTLA4 and PSCA showed stronger inhibition of tumor growth than the plasmid expressing PSCA alone. This result proved that elimination of the CTLA-4 inhibitory effect on the immune response *in vivo* can enhance the anti-tumor effect. In other words, blocking the immunosuppressive effect of CTLA-4 can stimulate the proliferation of immune cells, which can induce or enhance anti-tumor immune response.

These observations emphasize the potential of the CTLA4-fused DNA vaccine, which could represent a promising approach for tumor immunotherapy.
